# AMPKα1 deletion in fibroblasts promotes tumorigenesis in athymic nude mice by p52-mediated elevation of erythropoietin and CDK2

**DOI:** 10.18632/oncotarget.10687

**Published:** 2016-07-18

**Authors:** Yanhong Zhou, Hairong Xu, Ye Ding, Qiulun Lu, Ming-Hui Zou, Ping Song

**Affiliations:** ^1^ Center for Molecular and Translational Medicine, Georgia State University, Atlanta, GA 30303, USA; ^2^ Key Laboratory of Hubei Province on Cardio-Cerebral Diseases, Hubei University of Science and Technology, Xianning, Hubei 437100, China; ^3^ School of Medicine, Yangzhou University, Yangzhou, Jiangsu 225009, China

**Keywords:** AMPK, p52, erythropoietin, anchorage-independent cell growth, angiogenesis

## Abstract

Angiogenesis is essential for tumor development. Accumulating evidence suggests that adenosine monophosphate-activated protein kinase (AMPK), an energy sensor and redox modulator, is associated with cancer development. However, the effect of AMPK on tumor development is controversial, and whether AMPK affects tumor angiogenesis has not been resolved. We show that deletion of AMPKα1, but not AMPKα2, upregulates non-canonical nuclear factor kappa B2 (NF-κB2)/p52-mediated cyclin-dependent kinase 2 (CDK2), which is responsible for the anchorage-independent cell growth of immortalized mouse embryo fibroblasts (MEFs). Co-culture with AMPKα1 knockout MEFs (or their conditioned medium) enhances the migration and network formation of human microvascular endothelial cells, which is dependent on p52-upregulated erythropoietin (Epo). AMPKα1 deletion stimulates cellular proliferation of allograft MEFs, angiogenesis, and tumor development in athymic *nu/nu* mice, which is partly ameliorated by antibody-mediated Epo neutralization. Therefore, the AMPKα1-p52-Epo pathway may be involved in stromal fibroblast-mediated angiogenesis and tumorigenesis.

## INTRODUCTION

Adenosine monophosphate-activated protein kinase (AMPK) is a heterotrimeric Ser/Thr kinase complex that contains a catalytic subunit (α subunit) and two regulatory subunits (β and γ subunits) [1, 2]. Emerging evidence suggests that AMPK is strongly associated with cancer development [3], although the role of AMPK in cancer development and prevention is controversial [4]. AMPK is necessary for tumor development. For example, AMPKα is required for the tumorigenesis of H-Ras-transformed mouse embryonic fibroblasts (MEFs) in nude mice [5]. Recent work reported that AMPK activity is required and necessary for kinase suppressor of Ras 2 (KSR2)-mediated transformation and anchorage-independent growth of tumor cells MIN6 and NG108-15 [6]. Constitutive activation of AMPK induced by loss of the tumor suppressor folliculin is reported to augment hypoxia-induced factor (HIF)-mediated aerobic glycolysis, which is a Warburg metabolic transformation that enhances renal tumorigenesis [7]. Conversely, AMPK also has been implicated as a tumor suppressor. For example, AMPKα2 suppresses H-Ras^V12^-induced MEF transformation. AMPKα2 deletion enhances the tumorigenesis of H-Ras^V12^-transformed MEFs in the flank of athymic nude mice [8]. AMPKα2 is significantly reduced in breast cancer tissue samples, and re-expression of AMPKα2 in the human breast cancer cell line MCF-7 inhibits xenograft growth in athymic nude mice via p53 upregulation and cyclin D1 reduction [9]. Downregulation of AMPKα2 is associated with enhanced growth of liver cancer cells in mouse xenografts [10]. Loss of AMPKα1 in B cells accelerates c-Myc-driven lymphomagenesis via HIF-1α-mediated metabolic shift to aerobic glycolysis [11]. These combined results suggest that the reported discrepancy of AMPK function in cancer development may be due to different AMPKα isoforms and different experimental cancer cells and tissue systems. Therefore, the function of AMPK in cancer biology is not clearly understood, and the role of AMPK in tumor angiogenesis remains unclear.

Angiogenesis (the formation of new blood vessels) is a key mechanism that supports tumor development by providing nutrients and oxygen [12, 13]. Experiments in mouse models have clearly shown that fibroblasts in the stromal microenvironment have a significant role in tumor angiogenesis, which contributes to the initiation and progression of cancer [14–16]. Increases in smooth muscle α-actin (SM-α-actin)-expressing fibroblasts have been reported to enhance angiogenesis by recruiting endothelial progenitor cells [16]. However, the mechanisms by which AMPK controls fibroblast-mediated endothelial cell (EC) migration and differentiation are not fully understood.

AMPK negatively regulates nuclear factor-κB (NF-κB) signaling [17]. The upregulated non-canonical nuclear factor kappa B2 (NF-ĸB2) pathway strongly associates with tumor development and tissue hyperplasia. For example, mice with a C-terminal truncation of *Nfkb2* (leading to enhanced DNA binding of RelB/NF-κB2 p52 dimers) develop massive gastric hyperplasia and gastric outlet obstruction [18]. Adenoviral-mediated NF-ĸB2/p52 expression in LNCaP cells enhances tumor growth in intact male nude mice and induces tumor growth in castrated male nude mice, suggesting that NF-ĸB2/p52 overexpression induces androgen-independent growth of androgen-sensitive LNCaP cells [19]. However, whether p52 is involved in fibroblast transformation and tumor angiogenesis, as well as the underlying molecular mechanism is unknown.

Recent work showed that glycoprotein hormone erythropoietin (Epo) promotes breast tumorigenesis by activating JAK/STAT signaling in breast tumor-initiating cells (TIC) and promoted TIC self-renewal [20], although Epo is well known to regulate the production of red blood cells primarily by preventing apoptosis of erythroid progenitors [21]. Epo is reported to guide and enhance endothelial cell migration to initiate angiogenesis [22]. Currently, it is unclear whether or not p52 controls Epo, and Epo mediates tumor angiogenesis remain largely unknown. In the present study, we show that loss of AMPKα1 but not AMPKα2 activates NF-ĸB2, which upregulates CDK2 contributing to MEF transformation, as well as Epo leading to angiogenesis and tumorigenesis. These findings establish a new role for AMPKα1 in cellular transformation and stromal fibroblast-mediated tumorigenesis.

## RESULTS

### AMPKα1 deficiency confers anchorage-independent growth mediated by CDK2 induction

**Figure 1:**
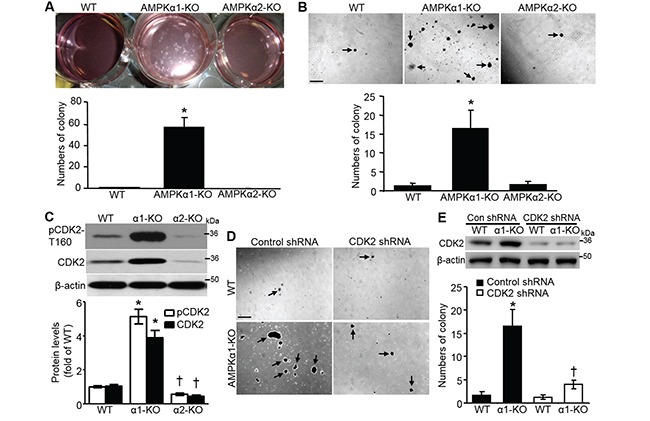
AMPKα1 deletion results in CDK2-mediated anchorage-independent MEF growth **A.** Spontaneous colony formation in AMPKα1-KO MEFs. Wild type (WT), AMPKα1-KO, and AMPKα2-KO MEFs (1 × 10^5^ cells/mL) were seeded and cultured on 6-well plates. Culture medium was changed every 2 days for 3 weeks. (Upper) Representative images showing colony formation of MEFs. (Bottom) Quantification of colony formation. *n*=5, *P<0.001 versus WT. **B.** Anchorage-independent cell growth assay (soft agar assay) of MEFs. (Upper) Representative images for colony formation. Scale bar =500 μm. (Bottom) Quantification of colony formation. *n*=10, *P<0.001 versus WT. **C.** Phosphorylated CDK2 at The-160 (pCDK2-T160) and CDK2 are upregulated in AMPKα1-KO MEFs. pCDK2-T160 and CDK2 protein in WT, AMPKα1-KO, and AMPKα2-KO MEFs were analyzed by Western blot (top). Quantification of pCDK2 and CDK2 data (bottom). *n*=4, *P<0.01 versus WT; ^†^ *P*<0.05 versus WT. **D.** Diminished anchorage-independent growth of AMPKα1-KO MEFs following CDK2 knockdown by shRNA. Representative images are shown. Scale bar =500 μm. **E.** Representative Western blot data indicate CDK2 knockdown by shRNA (top). Quantification of anchorage-independent MEF growth (bottom). *n*=4, *P<0.01 versus WT/control shRNA; ^†^*P*<0.01 versus α1-KO/control shRNA.

Proliferation of nontransformed cells is restrained by cell-cell contacts, which causes cells to exit the cell cycle and form a monolayer upon reaching confluency. The loss of contact inhibition is observed in the majority of cancer cell lines, and it is a hallmark of cellular transformation [12]. To assess the contribution of AMPKα to contact inhibition of MEF proliferation, we seeded wild type (WT), AMPKα1-knockout (AMPKα1-KO), and AMPKα2-KO MEFs at the same initial density (25% confluency) and allowed them to grow. As shown in Figure 1A, AMPKα1 deletion dramatically enhanced colony formation in MEFs cultured for 3 weeks, whereas either WT or AMPKα2-KO MEFs exhibited robust contact-dependent growth inhibition and formed a polarized quiescent monolayer after 3 weeks of culture. The results suggest that AMPKα1 deletion in MEFs leads to a loss of contact inhibition of cell proliferation. The soft agar assay confirmed that AMPKα1 deletion stimulated anchorage-independent growth (Figure 1B), which is in line with that AMPKα1 silencing rescues melanoma antigen (MAGE)-A3/6-RNAi-induced inhibition on colony formation of HeLa cells [23]. Cyclin-dependent kinase 2 (CDK2) is essential for anchorage-independent growth [24], so we analyzed the CDK2 profile. Both CDK2 and phosphorylated CDK2 at T160 were markedly elevated in AMPKα1-KO MEFs, whereas they were clearly reduced in AMPKα2-KO MEFs (Figure 1C). CDK2 knockdown by shRNA significantly inhibited anchorage-independent growth of AMPKα1-KO MEFs (Figure 1D and 1E), which may be due to the partial inhibition of cell proliferation. These results indicated that CDK2 was necessary for anchorage-independent growth of AMPKα1-KO MEFs.

### p52 mediates CDK2 upregulation

**Figure 2:**
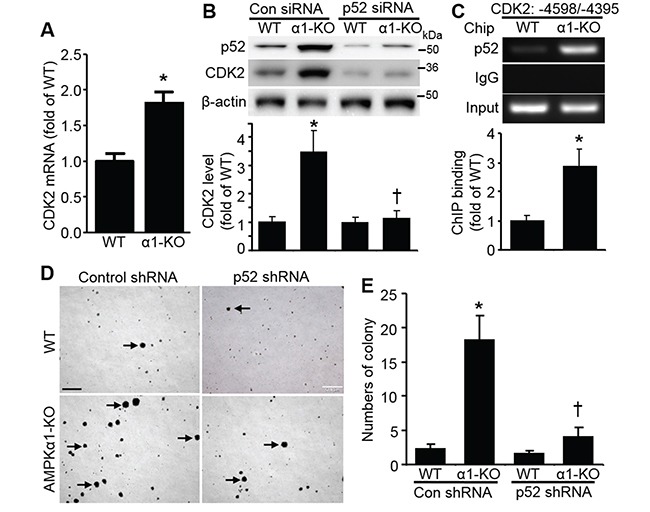
CDK2 elevation in AMPKα1-KO MEFs is mediated by p52 **A.** Upregulation of CDK2 transcription in AMPKα1-KO MEFs. CDK2 mRNA levels were measured by quantitative reverse transcription polymerase chain reaction in WT and AMPKα1-KO MEFs. *n*=5, *P<0.01 versus WT. **B.** p52 is responsible for CDK2 elevation in AMPKα1-KO MEFs. MEFs were transfected with either control siRNA or p52 siRNA (100 nmol/L) for 72 hours. Representative blot from three independent experiments (top). Quantification of Western blot data (bottom). *n*=3, *P<0.01 versus WT/control siRNA; ^†^*P*<0.05 versus α1-KO/control siRNA. **C.** Chromatin immunoprecipitation (ChIP) analysis of the CDK2 gene. MEF chromatin from WT and AMPKα1-KO mice was immunoprecipitated with anti-p52 or rabbit IgG as a negative control. Precipitated DNA or 10% of the chromatin input was amplified with gene-specific primers for mouse CDK2 promoter. This result is representative of four independent experiments. *n*=4, *P<0.05 versus WT. **D.** p52 knockdown by shRNA diminishes anchorage-independent growth of AMPKα1-KO MEFs. Representative images are shown. Scale bar =500 μm. **E.** Quantification of anchorage-independent MEF growth. Data are mean ± SD, *n*=5, *P<0.01 versus WT/control shRNA; ^†^*P*<0.01 versus α1-KO/control shRNA.

Next, we investigated the underlying mechanism for CDK2 upregulation in AMPKα1-KO MEFs. qRT-PCR analysis indicated that CDK2 mRNA was significantly elevated in AMPKα1-KO MEFs when compared with WT MEFs (Figure 2A). Knockdown of NF-κB2/p52 by siRNA dramatically abolished CDK2 elevation in AMPKα1-KO MEFs (Figure 2B). Chromatin immunoprecipitation (ChIP) assays further demonstrated that p52 bound to the CDK2 promoter (Figure 2C). p52 knockdown by shRNA eliminated anchorage-independent growth of AMPKα1-KO MEFs (Figure 2D and 2E).

### AMPKα1 deletion elevates NF-ĸB2/p52 via β-TrCP-mediated p100 processing

**Figure 3:**
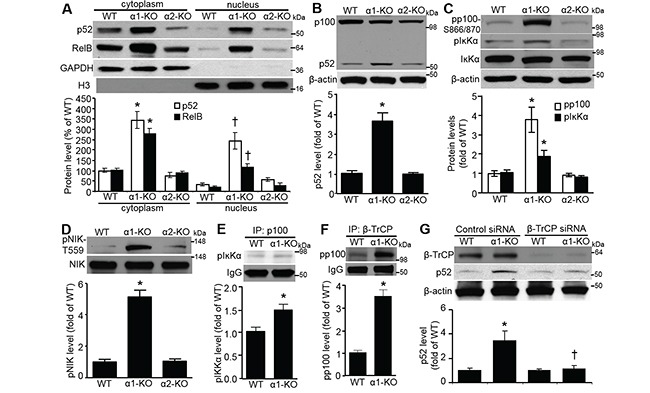
NF-κB2/p52 elevation in AMPKα1-KO MEFs occurs via β-TrCP-mediated p100 processing **A.** AMPKα1 deletion upregulates both p52 and RelB protein levels in both cytoplasm and nucleus of MEFs. p52, RelB, GAPDH, and histone H3 proteins in subcellular fractions of WT, AMPKα1-KO, and AMPKα2-KO MEFs were analyzed by Western blot (top). Quantification of the p52 and RelB data (bottom). *n*=4, *P<0.05 versus WT/cytoplasm; ^†^*P*<0.05 versus WT/nucleus. **B.** Levels of p100 and p52 proteins in WT, AMPKα1-KO, and AMPKα2-KO MEFs. p100 and p52 protein levels were assessed by Western blot analysis (top). This blot is representative of five blots from five independent experiments. Quantification of western blot data (bottom). *n*=5, *P<0.01 versus WT. **C.** Increased phosphorylation of p100 at Ser-866/870 (pp100-S866/870) and IκB kinase-α at Ser176 (pIĸKα) in AMPKα1-KO MEFs. pp100-S866/870, pIκKα, and IκKα protein levels were assessed by Western blot (top). This blot is representative of five independent experiments. Quantification of Western blot data (bottom). *n*=5, *P<0.01 versus WT. **D.** Elevated phosphorylation of NIK at T559 (pNIK-T559) in AMPKα1-KO MEFs. pNIK-T559 and NIK protein levels were assessed by Western blot (top). Quantification of Western blot data (bottom). *n*=5, *P<0.01 versus WT. **E.** Enhanced interaction of p100 with pIκKα in AMPKα1-KO MEFs. pIκKα-S176 was detected by immunoblotting after immunoprecipitation (IP) with anti-p100 (top). Quantification of Western blot data (bottom). *n*=5, *P<0.01 versus WT. **F.** Augmented association of β-TrCP with pp100 in AMPKα1-KO MEFs. pp100 was detected by immunoblotting after immunoprecipitation (IP) with anti-β-TrCP (top). Quantification of co-immunoprecipitation data (bottom). *n*=4, *P<0.01 versus WT. **G.** p52 elevation in AMPKα1-KO MEFs is mediated by β-TrCP. MEFs were transfected with either control siRNA or β-TrCP siRNA (100 nmol/L) for 72 hours. Representative blots from three independent experiments are shown (top). Quantification of Western blot data (bottom). *n*=3, *P<0.05 versus WT/control siRNA; ^†^*P*<0.05 versus α1-KO/control siRNA.

**Figure 4:**
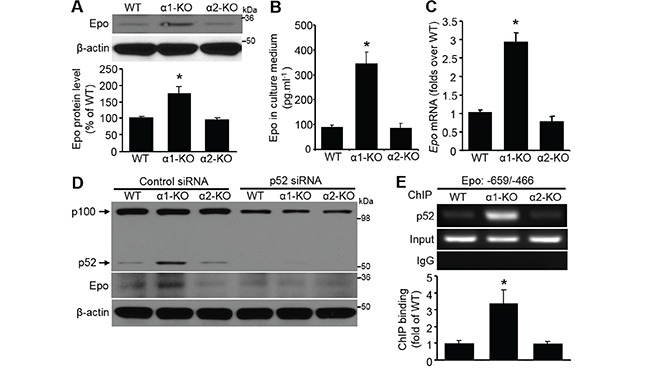
Elevated p52 is responsible for Epo induction **A.** Upregulated Epo in AMPKα1-KO MEFs. Epo protein level was assessed by Western blot analysis (top). Quantification of Western blot data (bottom). *n*=5, *P<0.05 versus WT. **B.** Epo was elevated in culture medium from AMPKα1-KO MEFs. Epo was concentrated from culture medium of MEFs with different genetic background and detected by ELISA. *n*=6, *P<0.01 versus WT. **C.** Upregulation of Epo transcription in AMPKα1-KO MEFs. Epo mRNA levels were measured by quantitative RT-PCR in WT, AMPKα1-KO, or AMPKα2-KO MEFs. *n*=5, *P<0.01 versus WT. **D.** Epo upregulation in AMPKα1-KO MEFs is mediated by p52. Three kinds of MEFs were transfected with either control siRNA or p52 siRNA (100 nmol/L) for 72 hours. Representative blots from three independent experiments are shown. **E.** Chromatin immunoprecipitation (ChIP) analysis of the *Epo* gene. MEF chromatin from WT, AMPKα1-KO, and AMPKα2-KO mice was immunoprecipitated with anti-p52 or rabbit IgG as a negative control. Precipitated DNA or 10% of the chromatin input was amplified with gene-specific primers for the mouse Epo promoter. This result is representative of four independent experiments. *n*=4, *P<0.05 versus WT.

We found that p52 and RelB protein levels in MEF cytoplasm and nucleus were elevated by AMPKα1 deletion (Figure 3A). As shown in Figure 3B, total p52 protein was profoundly upregulated in AMPKα1-KO MEFs compared with that in WT or AMPKα2-KO MEFs. AMPKα1 deletion substantially upregulated p100 phosphorylation at both Ser-866 and Ser-870 (pp100–S866/870); phosphorylation of this serine cluster in p100 creates a binding site for β-transducin repeat-containing protein (β-TrCP) [25]. P100 phosphorylation is crucial for the post-translational processing of p100 precursor into p52 [26, 27]. Accordingly, AMPKα1 deletion dramatically increased phosphorylation of IĸB kinase-α (IĸKα) at Ser-176 (Figure 4C); this is a well-known active form of Iĸkα [28], which is a key upstream kinase for p100 serines phosphorylation [29]. Further, AMPKα1 deletion enhanced the phosphorylation of NF-kappaB-inducing kinase (NIK) (Figure 3D), which may be due to the inhibition of protein phosphatase 2A (PP2A) [30]. Activated NIK preferentially phosphorylates IĸKα over IĸKβ and leads to the activation of IĸKα kinase activity [28, 31]. The associations of pIĸKα with p100 (Figure 3E), and pp100 with β-TrCP (Figure 3F), which is a critical E3 ubiquitin ligase for p100 ubiquitination and proteolytic processing [29, 32], were significantly enhanced in AMPKα1-KO MEFs. In addition, p52 induction in AMPKα1-KO MEFs was efficiently attenuated by siRNA technology knocking down β-TrCP (Figure 4G). Collectively, these results suggest that AMPKα1 deletion very likely activates NF-ĸB2 signaling, which is mediated by β-TrCP.

### Epo upregulation in AMPKα1-KO MEFs is mediated by p52

We reported previously that plasma Epo levels were conspicuously elevated in global AMPKα1-KO mice compared with that in WT mice [33]. Epo protein levels were significantly (*P*<0.05) higher in AMPKα1-KO MEFs than those in either WT or AMPKα2-KO MEFs (Figure 4A). The level of secreted Epo in medium from AMPKα1-KO MEFs culture was dramatically increased compared with that in WT or AMPKα2-KO MEFs culture medium (Figure 4B). Consistently, Epo mRNA levels were elevated in these cells (Figure 4C). Epo protein level was notably reduced by p52 siRNA, which dramatically downregulated the protein levels of both p100 and p52 (Figure 4D). ChIP assays confirmed that p52 bound to the Epo promoter area (Figure 4E). Collectively, these results suggest that AMPKα1 deletion induces p52-mediated Epo upregulation.

### Secreted epo from AMPKα1-KO MEFs stimulates endothelial cell migration and tube formation

**Figure 5:**
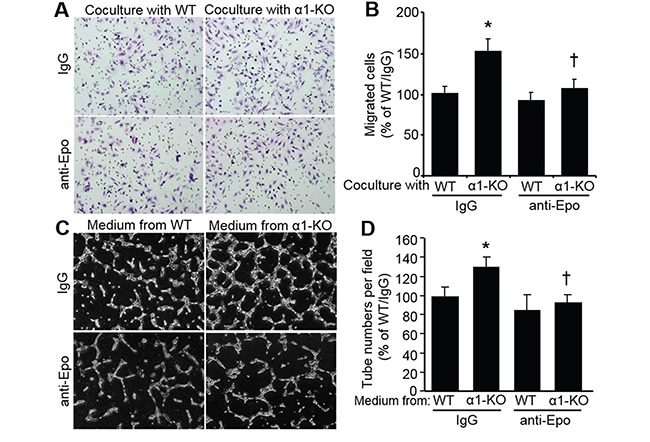
Secreted Epo from AMPKα1-KO MEFs enhances human microvascular endothelial cell (hmvEC) migration and tube formation **A.** AMPKα1-KO MEFs promote hmvEC migration, which is inhibited by Epo neutralization. Cell migration was measured using a Boyden chamber housing a polycarbonate filter in a 24-well plate assay system (8 μm pores, BD Biosciences). hmvEC (5×10^4^ cells/well in 500 μl of serum-free medium) seeded in upper chamber of Boyden chamber was co-cultured with WT MEFs or AMPKα1-KO MEFs seeded in bottom chamber plus nonspecific IgG or anti-Epo antibody for 16 h at 37°C. Cells on the lower surface of the filter were fixed with cold-methanol and stained with hematoxylin. Representative images are shown. **B.** Quantification of migrated cells. *n*=10, *P<0.05 versus WT/IgG; ^†^*P*<0.05 versus α1-KO/IgG. **C.** and **D.** AMPKα1-KO MEF-stimulated EC tube formation is ablated by anti-Epo neutralization. (C) Representative images showing tube formation. (D) Quantification of tube formation assay. *n*=4, *P<0.05 versus WT/IgG; ^†^*P*<0.05 versus α1-KO/IgG.

Epo is reported to increase EC migration [34]. Therefore, we analyzed the function of Epo in MEF-stimulated EC migration. Human microvascular endothelial cells (hmvEC) were co-cultured with mouse MEFs in a Transwell system. As shown in Figure 5A, AMPKα1-KO MEFs significantly (*P<0.05*) enhanced hmvEC migration compared with that of WT MEFs. We found that Epo neutralization with a specific anti-Epo antibody clearly blocked hmvEC migration enhanced by AMPKα1-KO MEFs (Figure 5A and 5B). Furthermore, hmvEC tube formation in Matrigel was markedly stimulated by conditioned medium from AMPKα1-KO MEFs compared with that from WT MEFs. However, Epo neutralization with a specific anti-Epo antibody attenuated tube formation enhanced by AMPKα1-KO MEFs (Figure 5C and 5D). These combined data strongly suggest that AMPKα1 deletion stimulates Epo expression in MEFs, which contributes to EC migration and network formation.

### AMPKα1-deleted MEFs lead to tumor formation in nude mice

**Figure 6:**
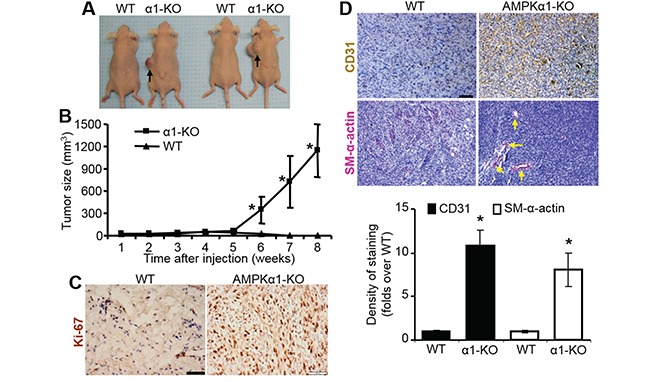
Allografed AMPKα1-KO MEFs promote tumor formation with increased vascularization in nude mice **A.** Representative images of tumors in nude mice inoculated with WT or AMPKα1-KO MEFs. *n*=8–10 per group. **B.** Tumor growth rate of AMPKα1-KO MEFs. WT MEFs or AMPKα1-KO MEFs mixed with 100 μl Matrigel were subcutaneously injected into nude mice and followed up for tumorigenesis as the indicated time. *n*=10–13 per group, Results are presented as mean ± SD. *P<0.01 versus WT. **C.** Staining of proliferation marker Ki-67 in allograft MEFs from nude mice. Allograft MEF tissues were collected from nude mice euthanized by carbon dioxide inhalation at 6 weeks after MEFs implantation. Representative images are shown. Scale bar =50 μm **D.** Allograft MEF tissues were stained with antibodies specific to CD31 or smooth muscle (SM)-α-actin. Allograft MEF tissues were excised from nude mice euthanized by carbon dioxide inhalation at 6 weeks after MEFs implantation. Representative images are shown (top). Scale bar =50 μm. Quantification of anti-CD31 or SM-α-actin staining (bottom). *n*=5–7 per group, *P<0.01 versus WT.

Subcutaneous implantation of immortalized MEFs from AMPKα1-KO embryos into immune-deficient nude mice dramatically promoted tumor development 6 weeks after implantation (Figure 6A). However, WT MEFs never induced tumor development in this setting (Figure 6A), only presented as the similar size or even shrunk size of original allograft during 6 weeks, consistent with a previous report [35]. The implanted MEFs did not grow at the beginning of week 5, but AMPKα1-KO MEFs then grew very quickly 5 weeks after MEF injection (Figure 6B). This may be due to the later development of blood vessels demonstrated by CD31 staining, which is a well-known endothelium marker [36] (Figure 6D), and by smooth muscle α-actin (SM-α-actin) staining, which is a well-established biomarker for vascular smooth muscle cells (VSMCs) [37] (Figure 6D). AMPKα1 deletion substantially enhanced MEF proliferation, as indicated by Ki-67 staining (Figure 6C). These data suggest that AMPKα1-deleted MEFs stimulate angiogenesis and subsequent tumor formation in nude mice.

### Epo elevation in AMPKα1-KO MEFs is required for tumorigenesis

**Figure 7:**
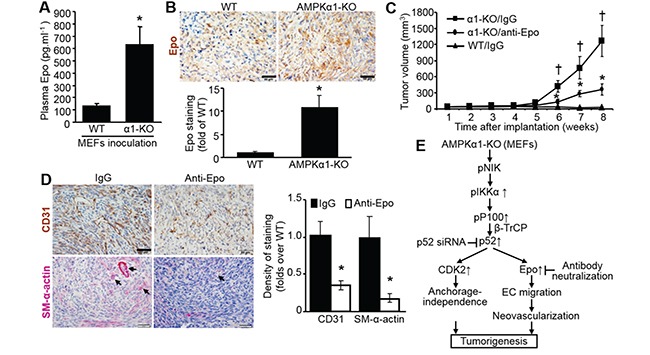
Epo elevation in AMPKα1-KO MEFs is required for tumorigenesis **A.** Plasma concentrations of Epo in nude mice inoculated with WT or AMPKα1-KO MEFs. *n*=10-13 per group; *P<0.01 versus WT. **B.** Epo upregulation in inoculated AMPKα1-KO MEFs in nude mice. Implanted MEF tissues were collected from nude mice euthanized by carbon dioxide inhalation at 6 weeks after MEFs implantation. Representative images show Epo staining (top). Quantification of anti-Epo staining (bottom). *n*=5 per group; *P<0.01 versus WT. **C.** Tumor growth rate of AMPKα1-KO MEFs in nude mice injected intraperitoneally (IP) with an antibody specific to Epo or IgG. *n*=8–10 per group, ^†^*P*<0.01 versus WT/IgG; *P<0.01 versus α1-KO/IgG. **D.** Representative images showing staining with antibodies specific to CD-31 or smooth muscle (SM)-α-actin (left). Scale bar =50 μm. Quantification of anti-CD31or SM-α-actin staining (right). Implanted AMPKα1-KO MEFs were collected from nude mice euthanized by carbon dioxide inhalation at the end of experiment (8 weeks after MEFs implantation). *n*=5–6 per group; *P<0.01 versus IgG. **E.** Mechanisms of AMPKα1deletion-stimulated anchorage-independent growth, neovascularization, and consequent tumorigenesis. AMPKα1 deletion in fibroblast activates NIK, which phosphorylates and activates IκKα, then enhance p100 phosphorylation recruiting E3 ubiquitin ligase β-TrCP, which facilitates p100 processing to p52. Upregulated p52 controls CDK2 and Epo expression. Epo-mediated neovascularization cooperating with CDK2-mediated anchorage-independent growth contributes to tumorigenesis *in vivo*.

As shown in Figure 7A, plasma Epo levels in nude mice inoculated with AMPKα1-KO MEFs were increased 5-fold compared with those in nude mice inoculated with WT MEFs. Epo staining was stronger in implanted AMPKα1-KO MEFs than in WT MEFs (Figure 7B). The tumor size in nude mice implanted subcutaneously with AMPKα1-KO MEFs was partially but significantly reduced by Epo neutralization with a specific anti-Epo antibody (Figure 7C). Epo antibody neutralization partly and clearly weakened the staining of both CD31 and SM-α-actin in the tumor area that developed after implantation of AMPKα1-KO MEFs (Figure 7D). These results imply that Epo-mediated angiogenesis or vascularization is an essential prerequisites for fast development of tumor.

## DISCUSSION

In this study, we demonstrated for the first time that deletion of AMPKα1 but not AMPKα2 enhances immortalized MEF transformation and tumorigenesis in nude mice. Mechanistically, we found that NF-ĸB2 p52 elevation in AMPKα1-KO MEFs was due to the increased NIK-mediated phosphorylation of IĸKα, which led to elevated p100 phosphorylation and enhanced the proteolytic processing of p100 precursor to p52 mediated by E3 ubiquitin ligase β-TrCP (Figure 7E). Elevated p52 levels upregulate CDK2, which contributes to the anchorage-independent growth of AMPKα1-KO MEFs. Increased p52 levels also upregulate Epo, which causes angiogenesis and subsequent tumorigenesis in athymic nude mice *in vivo* (Figure 7E).

Patient tumor samples, including lung squamous cell carcinoma, colon adenocarcinomas, and breast invasive carcinoma tumors expressing MAGE-A3/6, have decreased AMPKα1 protein levels [23]. In addition, as shown in The Human Protein Atlas (http://www.proteinatlas.org/ENSG00000132356-PRKAA1/cancer), lung cancer, stomach cancer, prostate cancer, and renal cancer tissues have negative staining for AMPKα1 protein compared with the corresponding normal tissues. While liver cancer, lung cancer, melanoma, and pancreatic cancer tissues show moderate AMPKα2 staining compared to respective normal tissues with negative staining (http://www.proteinatlas.org/ENSG00000162409-PRKAA2/cancer). However, there is no clinical report about AMPKα1 reduction/mutation in stromal cells in human cancer.

Oncogene H-RasV12-transformed WT MEFs dramatically develops tumors in nude mice [5], which is contrast to the results reported by Phoenix et al [8]. H-RasV12-transformed AMPKα1α2 double KO MEFs fails to form tumors in mice [5], which may be due to the increased cellular senescence in AMPKα2-KO MEFs [38]. Here, we showed that AMPKα1 deletion stimulated anchorage-independent MEF growth, which is consistent with a previous report that AMPKα1 deletion triggers MEF hyperproliferation and DNA damage [39]. These results further indicate that AMPKα1 may act as a tumor suppressor. Our results are in agreement with the results of previous reports. For example, AMPK activation suppressed tumor progression, whereas AMPKα1 inhibition/deletion promoted tumor formation, such as c-Myc-driven lymphoma development [11] and prostate cancer [40]. Conversely, several studies reported that AMPK or its activation was required for the formation of tumors such as astrocytic tumors [41] and experimental human breast tumors [42]. The reasons for these discrepancies are unknown. The reported discrepancy of AMPK effect in tumor/cancer development may be due to different AMPKα isoforms and different experimental tumor cells and tissue systems. The identification of additional regulators for AMPKα1 activation also will be of utmost importance.

Further, we demonstrated that AMPKα1 deletion triggered fibroblast transformation (anchorage-independent growth) via CDK2 upregulation (Figure 1D). In addition, we detected the protein levels of Merlin [43, 44] and E-cadherin [45, 46], both of them are associated with anchorage-independent growth. Merlin did not show difference among WT, AMPKα1-KO MEFs, and AMPKα2-KO MEFs (data not shown). E-cadherin protein level was dramatically decreased in both AMPKα1-KO MEFs and AMPKα2-KO MEFs as compared with WT MEFs (data not shown). Taken together, these data suggest that either Merlin or E-cadherin may not play the decisive role in AMPKα1 deletion-induced anchorage independence of cell proliferation. Moreover, AMPK activation by AICAR or metformin blocks the anchorage-independent growth of Lats1/2 double-knockout (DKO) MEFs by inhibiting the activity of Yes-associated protein (YAP) [47]. These results suggest that AMPKα1 regulates anchorage-independent growth via distinct molecules in different cell systems.

Epo is a cytokine that stimulates erythropoiesis; it is produced in the fetal liver and adult kidneys. The expression of Epo protein can be modulated by transcriptional regulators and post-translational modifications. Genetic evidence suggests that Epo is regulated at the transcriptional level by HIF1α at embryogenesis, or by HIF2α under physiological and stress conditions in adults [48, 49]. Platelet-derived growth factor (PDGF)-BB modulates Epo expression in stromal cells and perivascular cells via the transcription factor Atf3 [50]. Our results suggest that NF-ĸB2 p52 functions as a transcription factor for Epo expression in fibroblasts (Figure 4E). Epo exerts its biological functions via either canonical transmembrane Epo receptor (EpoR) [51] or the alternative Epo receptor, ephrin-type B receptor 4 (EphB4) [52]. Epo has been widely used for the treatment of cancer-related and chemotherapy-induced anemia [53]. However, it was reported to stimulate the growth or survival of tumors including ovarian tumor, breast tumor [52], and melanoma [54], and to promote tumor angiogenesis [50]. Our data indicate that Epo produced in fibroblasts promotes EC migration (Figure 5B) and angiogenesis in mice *in vivo* (Figure 7D), suggesting that clinical treatment of cancer patients with Epo should be approached with caution. In addition, it is interesting to investigate whether Epo promotes fibroblasts transdifferentiation into endothelial cells.

In summary, our results elucidate a previously unrecognized role of AMPKα1 deletion in loss of contact inhibition of cellular proliferation and angiogenesis, two key events in tumor/cancer initiation and progression. Therefore, the AMPKα1 pathway may be a promising therapeutic target for cancer treatment.

## MATERIALS AND METHODS

### Materials and reagents

The following antibodies were obtained from Cell Signaling Technology (Beverly, MA): rabbit anti-CDK2 (2546), rabbit anti-pCDK2-T160 (2561), rabbit anti-pp100-S866/870 (4810), rabbit anti-pIĸKα/β (2078), rabbit anti-IĸKα (2682), rabbit anti-NF-ĸB2 p100/p52 (4882), rabbit anti-NIK (4994), rabbit anti-β-TrCP (D13F10) (4394), and rabbit anti-PCNA (13110). β-TrCP siRNA (sc-37179), p52 siRNA (m) (sc-36043), CDK2 shRNA (m) lentiviral particles (sc-29260-V), NFκB p52 shRNA (m) lentiviral particles (sc-36043-V), goat anti-pNIK-T559 (sc-12957), rabbit anti-Epo (sc-7956), mouse anti-GAPDH (sc-32233), mouse anti-β-actin (sc-47778), were purchased from Santa Cruz Biotechnology (Santa Cruz, CA). Rabbit anti-Epo (ab65394) and rabbit anti-Ki-67 (ab15580) were purchased from Abcam (Cambridge, MA). Other chemicals and organic solvents of the highest available grade were obtained from Sigma-Aldrich. AMPKα1-KO and AMPKα2-KO mice were described elsewhere [55, 56]. Mice were handled in accordance with study protocols approved by the Institutional Animal Care and Use Committee of University of Oklahoma Health Sciences Center (Oklahoma City, OK).

### Cell culture, transfection, and infection

Primary MEFs obtained from 13.5-day old embryos of WT and knockout (KO) mice were immortalized by using a standard 3T3 protocol [57, 58]. The immortalized MEFs (referred to MEFs here) were maintained in Dulbecco's Modified Eagle's Medium (DMEM, 10-013-CV, Corning Cellgro) supplemented with 10% fetal bovine serum (FBS; Atlanta Biologicals), Glutamine, penicillin (100 units/ml), and streptomycin (100 mg/ml). Human microvascular endothelial cells (hmvEC) (Cat. # C-011-5C) were purchased from Life Technologies Corporation (Carlsbad, CA). The working concentration of siRNA duplexes applied was 100 nm. MEFs were transfected with target-specific siRNA or non-targeting siRNA for 72 h using Lipofectamine RNAiMAX (13778-150, Invitrogen) as previously described [59]. shRNA lentiviral particles transduction was performed in growth media supplemented with 8 ng/mL polybrene according to manufacturer's protocol.

### Protein extraction, immunoprecipitation, and immunoblotting

Whole cell extracts were prepared using cell lysis buffer (9803) from Cell Signaling Technology (Beverly, MA) with protease and phosphatase inhibitor cocktails (Cat. # 78440, Thermo Scientific). Immunoprecipitation was conducted using 900 μg of protein lysate. Protein samples (30–50 μg) were separated by SDS-PAGE, transferred onto nitrocellulose membranes, and probed with different primary antibodies as previously described [60, 61]. Following incubation with the appropriate horseradish peroxidase-linked secondary antibodies (Cell Signaling Technology), signal was visualized with an enhanced chemiluminescence detection system (GE Healthcare) and quantified by densitometry. Equal loading of protein was verified by immunoblotting with anti-β-actin or -GAPDH antibody.

### Epo enzyme-linked immunosorbent assay (ELISA)

One million MEFs were plated in 10-cm dishes in 8 ml of serum-free medium and cultured under normoxia (21% O_2_) for 16 hours. Cell culture media were spun at 2500 rpm to pellet down any cellular debris, and the supernatant was then collected and concentrated to 200 μl using Amicon Ultra-15 centrifugal filter units with Ultracel-10 membrane (UFC901024, EMD Millipore). Epo levels in cell culture media or blood plasma were determined in duplicate using a mouse Epo Quantikine ELISA kit (MEP00B, R&D Systems, Minneapolis, MN) according to the manufacturer's instructions.

### Soft agar assay

1.5 × 10^4^ MEFs were gently mixed in 2.5 mL of 0.4% SeaPlaque low melt agarose (Cat. #50101, Lonza) in complete DMEM culture medium as a soft agar on the top of 2.5 mL of 2% SeaPlaque agarose dissolved in PBS in 60-mm cell culture dishes. Once solidified at 4°C, the top cell/agarose mixture was covered with 2.5 mL of fresh medium, which was changed twice weekly. After 3 to 4 weeks of incubation at 37°C incubator, anchorage-independent growth in soft agar was quantified by counting the number of colonies (> 60 μm in diameter) in each field using a light microscope [62]. The experiments were replicated four times, and a representative set of data is photographed for presentation.

### Allograft studies

5×10^6^ MEFs with indicated genotypes mixed with 100 μL Matrigel (Cat. # 356237, BD Biosciences) were injected subcutaneously into the flanks of athymic nude (NU/NU, 7~8-week-old male) mice (Charles River, Wilmington, MA) [35]. IgG (554682) or anti-Epo antibody (554651, BD Pharmingen) was intraperitoneally injected (twice a week) since 4 weeks after MEFs implantation. Mice were checked for the appearance of tumors every week, and the tumor size was measured by external caliper. Tumor volumes (V) were calculated with the formula, V=L×W^2^×0.5, where L is the longest dimension and W is the shortest dimension of a tumor (mm) [63]. These studies were performed in full compliance with current Institutional Animal Care and Use Committee guidelines and requirements of University of Oklahoma Health Sciences Center. At 6 weeks after MEFs implantation or at the end of experiments with IgG or anti-Epo injection, animals were euthanized by carbon dioxide inhalation. Tumors were excised and individually weighed. Tumor tissue was frozen in liquid nitrogen for future studies or fixed with 4% buffered paraformaldehyde for paraffin embedding and sectioning.

### Immunohistochemical staining

Allografted MEFs were collected from athymic nude mice and fixed in 4% paraformaldehyde. The tissue samples were dehydrated and embedded in paraffin wax. Serial paraffin sections (4 μm) were obtained and kept at 37°C for more than 12 h. The sections were immersed in three consecutive washings in xylol for 5 min to remove paraffin, and then hydrated with five consecutive washings with alcohol in descending order 100, 100, 90, 80, 70% and deionized water respectively. The slides were immersed in citrate buffer solution (0.01M, pH 6.0) and heated for 30 min at 100°C, naturally cooled down and then immersed in 3% aqueous hydrogen peroxide for endogenous peroxidase ablation at room temperature for 20 min. Following steps were executed in a moist chamber. The sections were washed in PBS, quenched with blocking buffer (BioGenex, Fremont, CA). Sections were sequentially treated with individual primary antibody, secondary antibody (Dako, Carpinteria, CA) and DAB substrate (Dako, Carpinteria, CA). Finally, the tissue sections were counterstained with hematoxylin, dehydrated, cleared and mounted with neutral gums. In parallel, tissue specimens in which the primary antibody was replaced with blocking solution served as negative control.

### qRT-PCR

Confluent MEFs was washed with PBS and total RNA was extracted with TRIzol (Invitrogen). Total RNA (400 ng) from each sample will be used for cDNA synthesis, which will be performed using reverse transcription reagents from the iScript cDNA synthesis kit (Bio-Rad) according to the manufacturer's instructions and as described previously [64]. PCR will be performed using the following primers: mouse Epo forward primer, 5'-CCT CAT CTG CGA CAG TCG AG-3'; mouse Epo reverse primer, 5'-ACA ACC CAT CGT GAC ATT TTC T-3'; mouse CDK2 forward primer, 5'-CCT GCT TAT CAA TGC AGA GGG-3'; mouse CDK2 reverse primer, 5'-TGC GGG TCA CCA TTT CAG C-3'. Levels of Epo mRNA or CDK2 mRNA will be normalized to β-actin mRNA levels. Duplicate PCR reactions will be run for each sample, and results will be expressed as mean ± SD.

### Tube formation assay

The formation of vessel-like structures by hmvEC on growth factor-reduced Matrigel (BD Biosciences) was performed as previously described [65]. Briefly, 35-mm culture dishes were coated with Matrigel diluted (1:1) with supernatants from MEFs culture. The hmvEC were seeded on coated dishes at 3×10^5^ cells/dish and incubated at 37°C for 8 hours under normoxic conditions. In some dishes, either IgG or anti-Epo antibody was also added as indicated. Tube formation was observed using an inverted phase contrast microscope (Nikon, Tokyo, Japan). Images were captured with a videographic system (DEI-750 CE Digital Output camera; Optronics, Goleta, CA). The degree of tube formation was quantified by measuring the number of tubes in 30 randomly chosen low-power fields from each dish using the National Institutes of Health Image program. Each experiment was repeated 4 times.

### Chromatin immunoprecipitation (ChIP) assay

MEFs from WT, AMPKα1-KO, or AMPKα2-KO embryo will be subjected to ChIP using the ChIP Assay kit (Millipore Corporation, Billerica, MA). Briefly, 1% fresh formaldehyde will be used to cross-link proteins with DNA, and cells will be lysed in SDS lysis buffer. The cell lysate will be sonicated to shear the DNA into 120- to 400-bp fragments. Chromatin samples will then be pre-cleared with a salmon sperm DNA/protein A agarose-50% slurry for 30 min at 4°C and immunoprecipitated overnight with antibody against p52 (Ab7972, Abcam) or normal rabbit IgG as negative control. The Epo promoter region between -659 and-466 nucleotides will be amplified with the forward primer 5'-AAA GCA TCC GAG CCC CTT CTA GAC TC-3' and the reverse primer 5'-AGG GGG GTG CGC ATC TGA GAG AT-3'. The CDK2 promoter region between -4598 and -4395 nucleotides will be amplified with the forward primer 5'-TGG TGT TTT ATT TTG CTT GGG GTG GT-3' and the reverse primer 5'- GCA CAG TGT CAT GGG CCT AGA ATC C-3'. The amplified PCR products will be separated on a 1.5% agarose gel, stained with ethidium bromide, and visualized under UV light.

### Statistical analysis

Data are presented as mean ± SD. All studies will be conducted with a sufficient number of samples so as to obtain statistically robust results, with a confidence interval and *P*< 0.05. Paired *t*-tests will be used to compare results between two related groups. Comparisons between more than two groups will be performed using an analysis of variance (ANOVA) followed by the Bonferroni post hoc test using GraphPad Prism 5 program (GraphPad Software, La Jolla, CA).
